# Goat farm management and *Brucella* serological test among goat keepers and livestock officers, 2011–2012, Nakhon Si Thammarat Province, southern Thailand

**DOI:** 10.1016/j.onehlt.2016.08.001

**Published:** 2016-08-05

**Authors:** Thanidtha Te-Chaniyom, Alan F. Geater, Wandee Kongkaew, Usa Chethanond, Virasakdi Chongsuvivatwong

**Affiliations:** a Epidemiology Unit, Faculty of Medicine, Prince of Songkla University, Hatyai, Songkhla 90110, Thailand; b Faculty of Veterinary Science, Prince of Songkla University, Hatyai, Songkhla 90110, Thailand; c Veterinary Research and Development Center (Southern region), Thung Song, Nakhon Si Thammarat 80110, Thailand

**Keywords:** Goat farm management, *Brucella* serological test, Goats, Goat keepers, Livestock officers

## Abstract

Brucellosis, a zoonotic disease particularly affecting goats, emerged in Thailand in 2003, resulting in both an occupational hazard for goat keepers and livestock officers, and production losses. Farm management practices have been identified as risk factors associated with *Brucella* sero-positivity in many studies. Our finding in this study should be considered in order to strengthen the system of biosecurity control in farm animals as one health approach. The objectives of the study were to describe the distribution of potential risk factors by types of goat farms and to document the prevalence of human *Brucella* sero-positivity among goat keepers and livestock officers in Nakhon Si Thammarat, Thailand.

A cross-sectional study was conducted from September to December 2012. The study population included three types of goat farms: standard, community enterprise and private goat farms that were located in Nakhon Si Thammmarat Province in southern Thailand. Information on whether the farm had any *Brucella* sero-positivity goats since 2011 was retrieved from the local livestock office records. Information on farming management was also traced back to 2011. Field researchers collected information from goat keepers of the selected farms using a structured questionnaire. Goat keepers on all farms pre-identified (January to June 2012) as having had at least one positive goat were considered to have been exposed. Goat keepers on a random sample of farms having all goats with negative results were considered to be unexposed. Venous blood samples were collected from goat keepers exposed and unexposed and from livestock officers and the samples were tested by IgG ELISA. Statistical analysis was done under the complex survey design in R software.

Fourteen standard farms, 66 community enterprise farms and 68 private farms participated in the study; 82.4% (122/148) used public pasture and 53.4% (79/148) shared breeder goats with other farms. Farm management practices corresponding to pre-identified risk factors were more common in private farms. Large herd size (≥ 51 goats) and having dogs and/or rats on the farm were significantly associated with *Brucella* infection in animals (*P* < 0.05). Similar proportions of goat keepers in positive goat farm and livestock officers were positive for *Brucella* antibody (8.3% and 8.8% respectively).

Several goat farming management practices in the study area may increase the risk of *Brucella* infection in animals. Livestock officers in the area have a high risk of being infected with *Brucella*. Improving goat farm biosecurity practices in needed to reduce the risk of brucellosis in this area.

## 1 Introduction

**Fig. 1:**
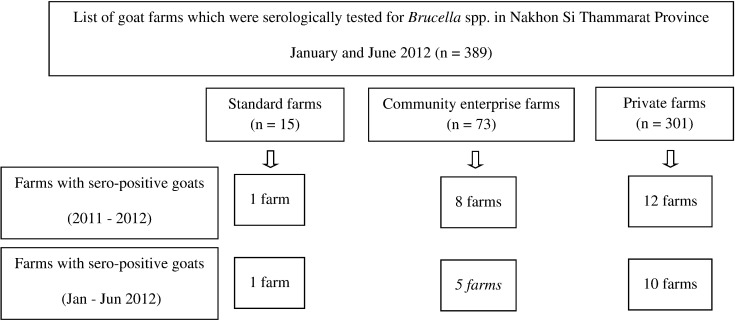
Sampling frame of the study stratified by type of goat farm and serological status in goat farms, Nakhon Si Thammarat Province, Thailand, 2011–2012.

Brucellosis is a zoonotic disease caused by a gram negative bacterium, *Brucella* spp. [4], [7]. In livestock, especially goats, *Brucella melitensis* (*B. melitensis*) is also the most common and can lead to mastitis, abortion and reduction of milk production [4], [7]. It has been estimated that 6 months after the introduction of *Brucella* infected animals into a herd, the infection rate can rise up to 50%–70% [7]. The organism can also infect humans and cause brucellosis, a systemic disease presenting with prolonged fever, which can be fatal if not treated [9]. For humans, *B. melitensis* is the most virulent species [4]. (See Fig. 1.)

In western and central parts of Thailand risk factors for *Brucella* sero-positivity in goat farms included herd size, close vicinity to other goat farms, grazing in communal pasture, having dogs on the farms, sharing buck with other farms, importing female breeder goats with an unknown source of *Brucella* infection and not using disinfectant [8], [17], [18], [21]. Sero-prevalence of goat *Brucella* infection in the southern part of Thailand during 2004–2006 was 1.47%, while in the western part it was 3.98% [2], [14].

In central Thailand, the first cases of human brucellosis emerged in 2003 [9], [10]. In southern Thailand, three human cases were diagnosed in 2004 during an abortion outbreak in goat farms [5]. Since 2003, human brucellosis cases have been reported nearly every year.

Brucellosis can be prevented by improving biosecurity practices in farms [4]. In Thailand, the Department of Livestock Development (DLD) promotes five principles of effective goat farming including farm attributes, farm management, herd health management, environmental management and animal welfare [6]. A goat farm registered as a standard farm with the DLD must follow the DLD guidelines. This biosecurity control in goat farm has been in effect since 2006. However, a community enterprise or private farm is not required to comply with these guidelines.

Based on concepts of “one health”, the current study tried to link animal health surveillance data with health of the human contact. The objectives of this study were to describe the distribution of potential risk factors by type of goat farm and to document the prevalence of human *Brucella* sero-positivity among goat keepers and livestock officers in Nakhon Si Thammarat, Thailand.

## 2 Methods

### 2.1 Study setting

The study was conducted in Nakhon Si Thammarat Province. The province ranks 8th among all provinces in goat population with 1677 goat farms and 18,807 goats registered with the DLD in 2011 [19]. This province also has the largest human population in southern Thailand with 1.5 million inhabitants [12]. The DLD has set up a specific disease surveillance and monitoring program focused on brucellosis and other diseases to track live goat movement across provinces. Since 2012, Nakhon Si Thammarat Province has established a brucellosis-free farm project. All goats in every farm with < 50 animals should be tested. A sample of goats will be tested if there are more than 50 goats. The Veterinary Research and Development Center (Southern region) (VRDC (Southern region)) uses the modified Rose Bengal test (100% sensitivity and 96.3% specificity on serum samples [8]). A serial test on Complement Fixation Test (CFT), recommended by the national guideline was omitted due to the shortage of materials for CFT at the VRDC (Southern region), only Rose Bengal Test (RBT) was available and used. Any positive goats should be culled under veterinarian officer supervision. During the following one month, another sample of goats should be tested until all goats are negative. At that time the farm should be tested twice a year.

### 2.2 Study population

Our cross-sectional survey was conducted in 17 of the 20 goat-raising districts of Nakhon Si Thammarat Province during September to December 2012. From January to June 2012, Nakhon Si Thammarat Provincial Livestock Office had tested for *Brucella* sero-positivity, on a total of 389 farms in these districts. These farms formed the sampling frame of the current study. The study goat farms were classified into 3 categories: 15 standard farms (certified by DLD); 72 community-enterprise farms (group of farms encouraged by the local livestock office but no certification.); and 302 private farms (not certified, but accessed by the local livestock office). Definition of diseased farm was a farm that had at least one sero-positive goat according to the data collected by questionnaire or records from the livestock offices.

### 2.3 Study samples

With small population of 15 standard and 72 community enterprise farms, they were all used in the study. On the other hand, of 302 private farms, 64 randomly selected were used. The sample size of the third group allowed 95% confidence interval of a risk factor, say, poor farm management, being ± 10% deviating from estimate value of 30% (finding from author pilot survey in a nearby province) For human subjects, the 389 farms in the database were re-stratified into farms with at least one *Brucella* sero-positive animal (16 farms) and the sero-negative farms (373 farms). All 16 farms in former group and 48 farms randomly selected from the latter group were visited. All goat keepers aged 7 years or older, who had stayed at the farm for at least 3 months were invited to join the study. All livestock officers who were responsible for brucellosis surveillance program, who working in the farms for at least two months were also invited to participated.

### 2.4 Data collection

Field researchers collected information from goat keepers using a structured questionnaire that had been piloted in a nearby province in southern Thailand where culture and the farm population were similar. Information on *Brucella* sero-positivity of the goat farms since 2011 was retrieved from the local livestock office records. Information on farm management was also traced back from the time of data collection (2012) to 2011. After completing the questionnaire, goat keepers of the selected farms, and livestock officers, were approached for venous blood samples. A 10 mL blood sample was taken from each consenting participant by a medical technician. All human sera were tested with IgG ELISA test kits (78% sensitivity and 98% specificity (EUROIMMUN)) to detect past *Brucella* infection with values of 22 RU/mL or above classified as positive.

### 2.5 Variables

The questionnaire focused on risk factor variables that were based on systematic reviews or biological plausibility for a role in disease transmission. These included farm characteristics (herd size, distance to the nearest farm, type of animals raised at nearest farm, and having dog/rat/cat on the farm), farm management (feed source, exposure to cattle or other goat herds, sharing goats, disease status of goat sources, quarantine process and using disinfectant) and abortion history.

### 2.6 Data analysis

Complete questionnaires were reviewed before coding. All variables on farm characteristics and herd management were cross-tabulated against farm type and then against whether or not the farm had at least one sero-positive animal. To represent the population of goat farms in Nakhon Si Thammarat, analysis were done based on the survey design. The weighting factor used in the analysis was the reciprocal of the farm sampling fraction within farm type, which was the stratification factor. Statistical significance level was set at < 0.05. Variables that showed some association with *Brucella* sero-positive (*P* < 0.2) in univariate analysis were included in an initial multivariate model. All statistical analysis was done using R software (version 3.0.1) and epicalc and survey packages.

### 2.7 Ethical consideration

All goat keepers signed a written consent form. From each consenting participant, a 10 mL blood sample was taken when the questionnaire was completed. Children aged < 18 years required consent from their parents. Children aged < 7 years were excluded. Persons who were pregnant or ill at collection time were excluded. Ethical approval was granted by the Ethics Committee of the Faculty of Medicine, Prince of Songkla University (EC: 55-198-18-5-2).

## 3 Results

Data cloud obtained from 14 of the 15 standard farms, 66 of the 72 community enterprise farms and 68 of the selected 71 private farms. Among the 10 missing farms we intended to used, eight had gone out of business and the owners of two farms were absent.

### 3.1 Farm component information

Problems related to risk factor variables were common in all three farm types. Of 148 goat farms, the goat keepers in 122 (82%) farms used public pastures for foraging and 118 (80%) had dogs and/or rats on the farms. Cattle were raised with goats in the same areas in 66% (95/145) of all three farm types, but this was more common among standard farms (12/13). Goats in the private farms were more likely to be in contact with goats from other herds (20/66). Appropriate quarantine processes were not practiced in 90% (34/38) of private and 78% (40/51) of community enterprise farms types, but were carried out by half of the standard farms (5/11). The private farms were more likely to be located close to other farms.

Variables that differed significantly among farm types included having other goat farms within 500 m, using the same raising field/pasture/farm area with others, introducing goats from unknown status, processing inappropriate quarantine, using disinfectant less frequently than once per month or never and having dogs and/or rats on the farms (Table 1).

**Table 1:** Goat farm characteristics and farm managements by farm registered type, Nakhon Si Thammarat Province, 2011–2012.

Variables	Type of farms n (%)			*P* value^⁎⁎^
	Standard (*n* = 14)	Community enterprise (*n* = 66)	Private (*n* = 68)	
Size of herd (*n* = 145)				
≥ 51 animals	3 (23.1)	9 (14.1)	5 (7.4)	0.159
Distance to the nearest goat farm (*n* = 143)				
< 500 m	4 (30.8)^a,b^	19 (29.7)^a^	36 (54.4)^b^	0.005
Distance from the nearest mammal livestock farm (Cattle, Pig, Buffalo) (*n* = 122)				
< 500 m	7 (50.0)	32 (60.4)	40 (72.7)	0.149
Type of the nearest livestock farm (n = 122)				
Cattle	13 (92.9)	50 (94.3)	52 (94.5)	0.970
Feed source from public pasture (*n* = 148)				
Yes	10 (71.4)	55 (83.3)	57 (83.8)	0.588
Raising cattle in the same goat area (farm/raising area) (*n* = 145)				
Yes	12 (92.3)	37 (56.1)	46 (69.7)	0.059
Other goat farm used the same raising field/pasture/farm area (n = 145)				
Yes	2 (15.4)^a,b^	7 (10.6)^a^	20 (30.3)^b^	0.011
Any aborted animals with the past 2 years (*n* = 148)				
Yes	5 (35.7)	24 (36.4)	26 (38.2)	0.957
Sharing breeder goat with other farm (*n* = 148)				
Yes	8 (57.1)	30 (45.5)	41 (60.3)	0.182
New goats from unknown *Brucella* infection source (*n* = 100)				
Yes	3 (27.3)^a,b^	11 (21.6)^a^	17 (44.7)^b^	0.038
Quarantine process (Separated pens and at least 7 days) (*n* = 100)				
Not/Inappropriate	6 (54.5)^a^	40 (78.4)^a,b^	34 (89.5)^b^	0.037
Using disinfectant (*n* = 148)				
< 1time/month or never	8 (57.1)^a,b^	34 (51.5)^a^	48 (70.6)^b^	0.045
Dogs and/or rats on the farm (n = 148)				
Yes	13 (92.9)^a,b^	57 (86.4)^a^	48 (70.6)^b^	0.023
Cat on farm (*n* = 148)				
Yes	8 (57.1)	29 (43.9)	36 (52.9)	0.490
*Brucella* serological status (2011 − 2012) (*n* = 148)				
Positive	1 (7.1)	8 (12.1)	5 (7.4)	0.567

(^⁎^ = For some variables the total sample is < 148 farms due to missing data).

(^⁎⁎^ = *P* value from Rao-Scott chi-squared test with complex survey analysis).

(^a,b^ = Value within rows not having a superscript in common differ significantly with *P* < 0.05).

### 3.2 Associated factors for *Brucella* sero-positivity in goat farm, 2011–September 2012

Univariate analysis showed large herd size (OR = 7.59, 95% CI = 1.57–36.74) and having dogs and/or rats on the farm (OR = 5.12, 95% CI = 1.04–25.21) were associated with *Brucella* sero-positive in goat farms (Table 2). Multivariate analysis found large herd size was the only significant risk factor (adjusted OR = 5.88, 95%CI = 1.19–29.15).

**Table 2:** Univariable analysis under complex survey design exploring factors associated with *Brucella* sero-positive in the goat farms, Nakhon Si Thammarat Province, 2011–2012.

Variables	Positive^†^ farms (n = 14) (%)	Total (n = 148)^⁎^ (%)	OR (95% CI)	*P* value^⁎⁎^
Size of herd				
≥ 51 animals	4 (30.8)	17 (11.7)	7.24 (1.52–34.39)	0.005^⁎⁎⁎^
Dogs and/or rats on the farm				
Yes	12 (85.7)	118 (79.7)	5.12 (1.04–25.21)	0.028^⁎⁎⁎^
Raising cattle in the same goat area (farm/raising area)				
Yes	9 (81.8)	95 (65.5)	4.56 (0.87–23.91)	0.052

(^⁎⁎^ = *P* value from Rao-Scott chi-squared test with complex survey analysis).

(^†^ = Positive meant farm had at least one sero-positive goat *Brucella* between 2011–September 2012).

### 3.3 Sero-prevalence of human *Brucella* infection in goat keepers and livestock officers

Of the 16 goat farms where sero-positivity for *Brucella* of the goats had been detected between January–June 2012, 24 farmers provided a serum sample (and average of 1.5 farmers per farm). Of these, 2 were positive (8.3%, 95% CI = 1%–27%). Among the 48 randomly selected sero-negative farms, 8 refused to participate. From the 40 consenting farms, 47 serum samples from the goat keepers were obtained (1.2 farmers per farm) and all were sero-negative. Seropositivity rates of the group were not significant different (Fisher's exact test *P* value = 0.11).

Among 34 livestock officers in 17 districts tested, 3 were positive (prevalence = 8.8%, 95% CI = 1.9%–23.7%). The past activities of the sero-positive officers included conducting vaccination, contacting placentas/vaginal secretions, contacting goat udders/milking, contacting carcass, blood collection, artificial insemination and drinking non-pasteurized goat milk. There was no significant association between any of these activities and seropositivity.

## 4 Discussion

The study revealed a high proportion of risk factors in goat farm practices across all types of farm in the area. These practices were more common among the private farms. The statistically significant risk factor for farm sero-positivity identified from this study was large farm size. For both exposed farm keepers and livestock officers the seroprevalence for *Brucella* spp. was < 10%.

Private goat farms are often clustered in the same community with shared resources. Previous studies in central Thailand revealed that 55% of meat goat farms in Nonthaburi Province used roughage from public areas and 32% borrowed bucks from other farms [21]. In a study in Chainat Province, 20.3% of meat goat farms also shared bucks [17]. In Portugal and Spain, 53.3% and 67.9% of small ruminant herds used communal pasture [3], [15]. All these statistics were lower than those of our study. In combination with the fact that our area is a major goat production region [19], these data indicate a need for improvement.

Despite poor farm management in southern Thailand, the sero-prevalence of animal *Brucella* spp. of 1% was relatively low compared to that in other regions such as western Thailand (11.5%) [8]. Over recent years, the disease prevalence has decreased because DLD started test and slaughter measures to control goat *Brucella* infection [11].

Serious human brucellosis cases among goat farmers were also reported in our region [5]. Our prevalence of sero-positivity among farmers was also lower than that reported in other regions of Thailand [8], [9]. This coincides with our area having smaller average herd size; 6.32 animals/household compared to 42.4 and 44.5 animals/household in the central and the western region Thailand respectively [13], [20].

Our and previous studies in Portugal, Jordan and Mexico consistently revealed large herd size to be a risk factor for farm *Brucella* infection [1], [3], [16]. A high animal density in the herd increases the opportunity for any animal contact and thus increases the chance of disease transmission [1]. Large herd size also raises the opportunity for new animals to be introduced from outside, thus increasing herd exposure to the disease [3]. Sero-positivity of the farm is established if at least one animal in the farm has a positive test. Assuming each animal in the population has an equal chance to be infected, the probability of having at least one positive would increase with the number of tests [16], or in this case, the herd size.

Our study did have certain limitations. Farms that had previously had two consecutive negative tests were not tested during the sampling period and therefore were not included in the study. In addition, the sero-positivity was based on modified Rose Bengal test alone; this test is known to have high sensitivity but not high specificity. So our reported prevalence was biased towards a high value. Farm classification was based on registration, which was voluntary. Also, some farms could be classified into both a standard and community enterprise farm type. In our analysis these farms were treated as standard farms because the farm management practice should be following DLD criteria. The serological tests are likely to have had a certain degree of misclassification, which was unavoidable. The sample size of goat keepers was small. Thus the power to detect associations with human sero-positivity was limited.

In conclusion, there were many farm practices that were inappropriate for controlling or preventing the spread of *Brucella* sero-positivity in the study area. Sero-positivity in goats among goat farms was associated with a large-herd size. *Brucella* sero-positivity is an occupational hazard among livestock officers and goat farmers [9].
